# A bibliometric and visualization analysis of global research status and frontiers on autophagy in cardiomyopathies from 2004 to 2023

**DOI:** 10.1097/MS9.0000000000002595

**Published:** 2024-09-25

**Authors:** Tao Bao, Yucheng Zhang, Yuxia Yang

**Affiliations:** a Chun'an County First People's Hospital, Hangzhou P. R. China; b Changshu Hospital Affiliated to Nanjing University of Chinese Medicine, Suzhou, P. R. China; c Northern Jiangsu People's Hospital, Affiliated to Yangzhou University, Yangzhou P. R. China

*Dear Editor*,

As we all know, *International Journal of Surgery* is a well-received and top academic journal in the field of surgery, and we are keen to read the high-quality research in the journal, among which a study titled ‘A bibliometric and visualization analysis of the global research status and frontiers on autophagy in cardiomyopathies from 2004 to 2023^1^’ caught our attention. This study used scientific literature search and econometric analysis to analyze the research hotspots and trends in autophagy in cardiomyopathies, obtained keyword information for these studies and summarized the key countries, institutions, authors, and journals, which provide important references for scholars needing to know more about this field.

As mentioned in the authors’ article, cardiomyopathies are a major health problem of concern and a huge economic burden on society, fully suggesting the need for this study. However after our careful reading, we have some suggestions on some parts of this study, especially the process of data handling, which we would like to discuss with the authors and the general readers. The source of data is a primary and important step in the bibliometric analysis, and if the articles included in the study in the first step do not fulfill the expectations, the subsequent results will be highly biased.

The first concern is that the retrieval formula provided by the authors in the Methods section, we were unable to reproduce the results mentioned by the authors by searching through WOSCC, and the authors should have re-validated the retrieval strategy. Secondly, the authors mentioned in the data retrieval section that ‘The exclusion criteria were as follows (1) the topic was not related to the role of autophagy in cardiomyopathy; (2) the article was a conference abstract, news, or briefing paper’, the authors claimed to exclude literature whose subject matter was not related to the role of autophagy in cardiomyopathy, yet this process is not reflected in the flowchart, which simply excludes studies outside of the year and conference abstracts, news, or briefs. The authors’ search strategy still incorporates more irrelevant literature, which is a common problem, and the incorporation of too much irrelevant literature can lead to biased findings; however, the authors have neglected the process of quality control in the methods section. Authors should conduct the critical step of data cleaning in detail, how to screen and clean the studies that are irrelevant to this study, and even the keywords or organizations have diversity in the way they are used, such as synonyms, which authors should fully take into account in the process of data cleaning.

In addition, the authors mention the use of the following scheme in their search strategy. ‘In this study, we searched the literature related to autophagy in cardiomyopathy from Web of Science Core Collection (WoSCC) for the last 20 years on January 18, 2024 and performed a comprehensive analysis using bibliometric methods’. As we know, the Web of Science Core Collection (WOSCC) is a collection of databases that integrates several fields, including the Science Citation Index Expanded (SCIE), which includes research in the natural sciences from 1999 to the present; SSCI (Social Science Citation Index), which includes research in the social sciences from 2005 to the present; AHCI (Arts and Humanities Citation Index), which includes research in the arts and Humanities from 2005 to the present; AHCI (Arts and Humanities Citation Index) for research in the arts and humanities from 2005 to the present; ESCI (Emerging Sources Citation Index) for research resources that have emerged in recent years; CCR-Expanded (Current Chemical Reactions) for information on the latest chemical reactions from 2019 to the present; and IC (Index Chemicus) for information on organic compounds from 1993 to the present. The authors’ current research topic was biomedical-related content, and we still recommend that the authors include the larger database Scopus, as well as the biomedical literature databases MEDLINE, PubMed, and the Cochrane Library, and that the content of the various databases be summarized and screened for comprehensiveness of the included literature^2,3^. This paper does not describe the search strategy accurately enough, the authors of this research limited the literature type to Article and Review, as far as we know most of the bibliometric analysis of the content of the bibliometric analysis only choose Article to analyze^4,5^, because the review paper is just a synthesis of the discussion and analysis of the previous articles, so we suggest that the authors would be better off to choose the appropriate type of literature and a more precise description in order to improve the scientific and rigor of the research results.

Finally, there are some details that do not affect the big picture that the authors have inaccurately described in some places in this paper, and we suggest that the authors continue to improve them in subsequent studies. For example, the authors claimed in the Methods section that the search included the period from January 1, 2004 to December 31, 2023, whereas Figure 1 included the year 2024, and the year 2024 had citations on the basis of no number of documents, which the authors should have verified; the authors’ legend of Figure 6B did not match the content of the picture, and the picture did not show the network of the top 10 authors only; there was an inconsistency between the manuscript text and there are inconsistencies in the text of the image, where the text in the manuscript is in upper case or all caps and the text in the image is in lower case or abbreviated, and the authors should follow up to harmonize these.

In conclusion, we congratulate all authors for the successful completion of this bibliometric work, which is the first bibliometric and visualization analysis of autophagy in cardiomyopathies. In order to further improve the scientific validity and rigor of the study, we believe that the recommendations we present here can help the authors to build on their previous work in a more reliable and comprehensive manner.

## Ethical approval

Not applicable.

## Consent

Not applicable.

## Source of funding

This research was supported by the Postgraduate Research & Practice Innovation Program of Jiangsu Province(Grant SJCX24_2351).

## Author contribution

Y.Y.: made a great contribution to the overall research, design, and conception; T.B.: wrote manuscripts for data integration processing; Y.Z.: data collection.

## Conflicts of interest disclosure

The authors declare no conflict of interest, financial or otherwise.

## Research registration unique identifying number (UIN)

Not applicable.

## Guarantor

Corresponding author Yuxia Yang was able to take full responsibility for the study and make all decisions.

## Data availability statement

The data used to support the findings of this study are available from the corresponding author upon request.

## Provenance and peer review

This paper has not been published anywhere or is a repeat submission.
